# Ischemia-Reperfusion Injury in Peripheral Artery Disease and Traditional Chinese Medicine Treatment

**DOI:** 10.1155/2021/4954070

**Published:** 2021-12-02

**Authors:** Zenghui Liang, Wentao Zhang, Tianyi Zhu, Yunsong Li, Pengkai Cao, Yanping Wu, Yanrong Zhang

**Affiliations:** ^1^The Third Hospital of Hebei Medical University, Shijiazhuang, China; ^2^Department of Gland Surgery, Second Hospital of Hebei Medical University, Shijiazhuang, China; ^3^Clinical Laboratory, The Third Hospital of Hebei Medical University, Shijiazhuang, China; ^4^Department of Vascular Surgery, The Third Hospital of Hebei Medical University, Shijiazhuang, China; ^5^Department of Pediatric Ophthalmology, Cangzhou Central Hospital, Cangzhou, China

## Abstract

Peripheral artery disease (PAD) is a serious public health issue, characterized by circulation disorder of the lower extreme that reduces the physical activity of the lower extremity muscle. The artery narrowed by atherosclerotic lesions initiates limb ischemia. In the progression of treatment, reperfusion injury is still inevitable. Ischemia-reperfusion injury induced by PAD is responsible for hypoxia and nutrient deficiency. PAD triggers hindlimb ischemia and reperfusion (I/R) cycles through various mechanisms, mainly including mitochondrial dysfunction and inflammation. Alternatively, mitochondrial dysfunction plays a central role. The I/R injury may cause cells' injury and even death. However, the mechanism of I/R injury and the way of cell damage or death are still unclear. We review the pathophysiology of I/R injury, which is majorly about mitochondrial dysfunction. Then, we focus on the cell damage and death during I/R injury. Further comprehension of the progress of I/R will help identify biomarkers for diagnosis and therapeutic targets to PAD. In addition, traditional Chinese medicine has played an important role in the treatment of I/R injury, and we will make a brief introduction.

## 1. Introduction

Peripheral artery disease (PAD) is defined as the obstructing or narrowing of the arteries of low extremities due to atherosclerotic plaque, subsequently restricting or blocking blood flow to the affected lower extreme. The PAD is characterized by the reduced oxygen and energy delivery to lower limbs, resulting in exertional leg pain that limits the ability of walking, which would be resolved through rest. If limb ischemia is severe, it may cause pain on rest or amputation [1]. Since atherosclerosis is a systemic disease, a portion of patients with PAD will have heart or cerebrovascular disease [2]. The most risk factors of PAD are consistency with myocardial infarction and stroke, which indicated that PAD is an independent risk factor [3, 4]. Patients with short-distance claudication or severe ischemia undergo revascularization to restore blood, which prevents limb pain at rest and limb amputation. Nevertheless, PAD is still a serious health hazard problem with significant morbidity and mortality. Further understanding of physiopathology needs further research to improve therapeutic strategies.

**Figure 1:**
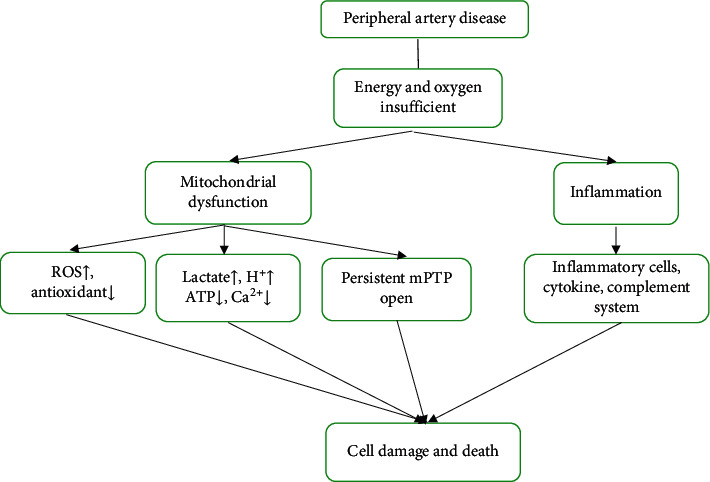
The pathogenesis of PAD. PAD is characterized by reduced oxygen and energy delivery to lower limbs, and undergoing revascularization would restore blood. However, this surgery would induce I/R injury. The mechanisms of I/R injury are multifactor, mainly consisting of mitochondrial dysfunction and inflammation.

Ischemia-reperfusion (I/R) is characterized as the reduction of blood supply to the tissue or organ, which subsequently leads to vascular restoration and concomitant reoxygenation of downstream tissue [5]. The restriction of oxygen supply leads to insufficient metabolic supply, causing tissue hypoxia. Contrary to expectations, restoration of blood and oxygen is associated with aggravation of injury and promotion of inflammation. The pathophysiology of I/R injury is various (Figure 1). Mitochondrial dysfunction can reduce energy supply and oxidative stress, and inflammation may result in intermittent claudication, limb pain at rest, and amputation. Reactive oxygen species (ROS) derived radicals such as superoxide anion (O_2_^−^), hydrogen peroxide (H_2_O_2_), hydroxyl radical (-OH), hypochlorous acid (HOCI), and nitric oxide-derived peroxynitrite. ROS are potent oxidizing property, causing cell membrane damage by lipid peroxidation, which is responsible for local and systemic damage caused by I/R injury. In addition, inflammation plays an important role in I/R injury. Depending on the degree and the duration of ischemia of the affected organ, it can trigger remote complications such as the heart and kidney [6, 7].

Traditional Chinese medicine (TCM) has been used in the treatment of various diseases for more than 2000 years. Several studies have shown that TCM can be used in the treatment of ischemia and I/R injury through different mechanisms, including regulation of energy metabolism, inhibition of antioxidants, and reduction of inflammatory cytokines. This review covers the main mechanisms of skeletal I/R injury, and the application of TCM in I/R therapy is introduced, which may provide a theoretical basis and novel idea for dealing with I/R injury of PAD.

## 2. Mitochondrial Dysfunction

Mitochondria participate in multiple physiological functions, including energy metabolism, Ca^2+^ signal, cell differentiation, and apoptosis [8–10]. During recent years, many studies have described the mitochondrial functions in I/R injury. The reduction of blood supply causes insufficiency of oxygen and affects the function of the electron transport chain. Skeletal muscle is energy dependent, mainly provided by mitochondrial metabolism. With increased ATP turnover, the skeletal muscle transforms from rest to activity, and the substrates of energy production can be oxidized [11]. The I/R injury of the lower limb affects the local muscle environment through various processes, resulting in the reduction of muscle function . The sensibilities of different muscles are discrepant due to their antioxidant capacities [12, 13]. PAD-induced I/R injury causes myopathic and neuropathic changes [14], which may also impair the function of mitochondria [15, 16].

ATP is mainly produced by the oxidative phosphorlation process in resting myocyte. Sestrin2 functions as a scaffold protein, which interacts with OXPHOS components to keep mitochondrial integrity under I/R stress [17]. The producing substrates of energy include phosphate compounds, glucose, glycogen, and lipids in mitochondria. In addition, mitochondria are the major source of ROS. Both complexes I and III of the mitochondrial respiratory chain can produce ROS. The main reactive species are superoxide anion and nitric oxide (NO), subsequently forming secondary reactive species, such as H_2_O_2_ and peroxynitrite [18]. Therefore, the mitochondria play an important part in the skeletal muscle fiber physiology, both in energetic metabolism for energy supply and cell signaling.

Ischemia in the lower extremity restricts the nutrient and oxygen supply, leading to a mass of ionic and metabolic changes. Since oxygen is lacking, mitochondrial OXPHOS can be affected, and the potential of the mitochondrial membrane decreased [19]. The activities of the electron transport chain (complexes I, II, and IV) are changed during ischemia [20, 21], which lead to reduced synthesis of ATP and elevation in concentrations of inorganic phosphate and adenine nucleotide [22, 23]. In the progression of ischemia, ATP is catabolized into xanthine and hypoxanthine; subsequently, the substrates conduce to ROS production during the progression of reperfusion [22, 24]. For continuously providing energy, anaerobic metabolism and phosphocreatine pathways are activated to generate ATP. ATP, phosphocreatine, and glycogen exhaust within 7 hours, which correlates with skeletal muscle death [23, 25–28]. These changes of metabolism cause accumulation of H^+^, nicotinamide adenine dinucleotide (NAD), and lactate, which make extra- and intracellular acidified. [6, 29–32]. Then, the Na^+^/H^+^ exchanger is activated to recover H^+^. Various ionic exchangers of the sarcolemma are restrained by low ATP, including Na^+^-K^+^-ATPases and Ca^2+^-ATPases. And Na^+^-Ca^2+^ antiporters are reversed to recover the Na^+^ concentration, subsequently resulting in the accumulated concentration of Ca^2+^ [6, 33]. The accumulation of Ca^2+^ causes damage of cell integrity by degrading lysozymes, proteases, and nucleases and induces inflammation and cell death through necrosis and apoptosis [34, 35].

ROS are one of the main factors responsible for local and systemic damage in the progress of I/R injury. ROS include O_2_^−^, H_2_O_2_, -OH, HOCI, and NO-derived peroxynitrite. During the progress of ischemia, ROS are mainly produced by complexes I and III of the mitochondrial respiratory chain [32, 36, 37]. Several enzymes play an important role in the production of ROS, such as the xanthine oxidase (XO) system, NADPH oxidase (NOX) system, and nitric oxide synthase (NOS) system.

XO system includes XO and xanthine dehydrogenase (XDH). XO plays a primary role in ROS production, which localizes in macrovascular endothelial cells of the skeletal muscle. In the ischemic state, XDH is converted into XO because of the low ATP level, and XO may induce the production of ROS during the conversion of hypoxanthine to uric acid. NOX enzymes promote the production of superoxide and hydrogen peroxide, and NOS uncoupling leads to the generation of ROS; both processes may induce I/R injury. These ROS damage membranes, including those of the mitochondria. Damaged mitochondrial membranes lead to the release of caspases and activation of apoptosis. In addition, hypoxia increased the activity of NOS forming NO, which reacts with superoxide to give peroxynitrite that damages nucleic acids and lipids. Meanwhile, defense systems can reduce ROS-induced damage, which include catalase, glutathione peroxidase (GPx), superoxide dismutase (SOD), glutathione, coenzyme Q, and vitamin E. However, in the PAD muscle, matrix SOD has been demonstrated to be deficient, which is the initial line of ROS defense in mitochondria.

Sufficient oxygen supply in the reperfusion progression is the primary reason of myocyte death via generating excessive quantities of ROS. Production of ROS in mitochondria is a self-amplified process. This process is hard to eliminate as the antioxidant defenses are also changed by ischemia. Meanwhile, ischemia and reperfusion can further affect the activity of mitochondrial complexes I, II, III, and IV, which affects the membrane channel and increases cytosolic Ca^2+^ concentration [24, 37, 38]. Elevated Ca^2+^ concentration stimulates proteases and phospholipases, which affect membrane receptors, ion channels, and enzymes, leading to cell membrane degradation and decreasing cell survival rate [39, 40]. Furthermore, osmotically active molecules accumulate and recover in cells generating an osmotic gradient within intra- and extracellular environments, which causes cells to water uptake, swelling, and break up [23]. It was reported that improving mitochondrial quality control is critical to improve the effectiveness of current treatments in PAD such as exercise [41]. Therefore, alleviation of oxidative stress may be a useful strategy to deal with I/R injury. And inhibition of the XO system, NOX system, or NOS system may be a feasible method. In addition, increasing endogenous antioxidants can directly regulate ROS, which may alleviate I/R injury.

In order to maintain normal physiological function, mitochondria are constantly changing dynamically, which is called mitochondrial dynamics. Mitochondria dynamics involves mitochondrial fusion, fission, and autophagy, which plays an important role in maintaining cellular physiological function and hemostasis. Studies indicated that mitochondrial dynamics changed during I/R injury. Mitochondrial fusion helps mitigate stress by mixing partially damaged mitochondria. And fission is needful to create new mitochondria. However, during overloaded cellular stress in some diseases, including I/R injury, fission may facilitate apoptosis [42].

The mitochondrial permeability transition pore (mPTP) is located in the inner mitochondrial membrane. The mPTP is a nonselective multiprotein channel, which can be regulated by various cell factors, such as ROS, ATP, inorganic phosphate, pH, Ca^2+^, and membrane potential. The biochemical changes during I/R injury can turn up the mPTP. The persistent mPTP opening deregulates the release of matrix Ca^2+^, restricts OXPHOS, swells the matrix, and eventually ruptures the outer membrane by the release of apoptotic proteins and cell death. Meanwhile, the opening of mPTP can promote the production of ROS.

## 3. Inflammation

I/R injury is associated with the activation of inflammation and immune system. The characteristic of reperfusion injury is immune responses, including natural antibody recognition of neoantigens and activation of the complement system. I/R induced by PAD occurs in a sterile environment, which has been termed sterile inflammation. Sterile inflammtion shares similar response to those ivoked by microorganism.. The sterile immune response, through pattern recognition molecules such as toll-like receptors (TLRs), activates immune cells. Ligand binding to TLRs activates downstream signaling pathways, subsequently inducing the generation of proinflammatory cytokines and chemokines [43]. During I/R, with the cell damage and death, endogenous molecules can activate these receptors. And ligands are termed damage-associated molecular patterns (DAMPs). DAMPs are normally located in intracellular, they will release to extracellular at the time of tissue damage [44, 45]. The function of DAMPs is that they activate immune response, restrict harmful immune response, and promote tissue integrity [46, 47]. TLR4 is one of the famous pattern recognition receptors, which mediates inflammation through its activation by lipopolysaccharide. Oxidative stress can enhance the activation of TLR4 [48]. Deletion of TLR4 is hyporesponsive to lipopolysaccharide [49]. Antagonists for TLR4 or regulators which reduce TLR4 expression may be a useful treatment.

During I/R, accumulation of inflammatory cells has been found. These inflammatory cells include monocytes, dendritic cells, and granulocytes [50–53]. The role of inflammatory cells is not fully studied. They may activate inflammation and accelerate tissue injury or restrict the recovery of injury [54].

The benefited function of inflammatory cells depends on their production. For example, dendritic cells may produce inflammatory cytokine interleukin-10 (IL-10) [55, 56]. They can downregulate the expression of tumor necrosis factor-*α* (TNF-*α*), IL-6, and ROS. Nearly, all inflammatory cells express NADPH oxidase contributing to format ROS and peroxynitrite. Peroxynitrite may induce oxidative DNA injury and activate nuclear enzyme poly (ADP-ribose) polymerase-1 (RARP-1). Granulocytes are involved in tissue repair. Howerver, if they are accumulated enough, they may lead to uncontrolled inflammation and tissue injury [57]. In addition, I/R injury inducesadaptive immune response, which involves various T lymphocytes. The function of T lymphocytes needs further research in PAD-induced I/R.

The complement system is a biological cascade and promotes clear pathogens from the organism. It acts as an immune surveillance system, which can discriminate healthy host tissue, apoptotic cells, foreign intruders, and cellular debris [58]. In the progress of I/R injury, the complement system is activated. It was confirmed that ischemia upregulates the expression of the antigen on cellar surfaces, which binds to the IgM natural antibody. Natural antibodies are a major component of B1 cells, which produce IgM and IgG [59]. Antigen-antibody complex causes C1 binding, complement activation, and formation of C3a and C3b. Subsequently, C3b activates a complement cascade causing to form a membrane attack complex (MAC). The MAC can stimulate macrophages to release prostaglandin E2, and neutrophils release ROS, IL-1, etc [59–61]. Studies showed that inhibiting the component of the complement could be an effective treatment of I/R injury, but it needs further verification [62–64].

Platelet aggregated excessively and platelet-derived mediators aggravate injury during I/R. Endothelial interactions activate platelets [65]. Subsequently, the platelets transport to the sites of injury. In addition, I/R promotes coagulation [17]. It was reported that several anticoagulants can inhibit clot formation [66, 67], such as tissue factor inhibitor, protein C, and antithrombin heparin. Besides, cytokines are factors that transmit signals between cells and include various and numerous families of polypeptide regulators. They can play a role in immunomodulating. It was verified that IL-1, IL-6, thromboxane A2, and necrosis factor are referred to I/R injury.

In conclusion, inflammation is important progression, which may cause cell damage and repair. It inhibits the activation of the complement system and reduces proinflammatory cytokines; chemokines are a potential therapeutic strategy to reduce tissue damage, induced by I/R injury.

## 4. Cell Damage and Death in I/R Injury

**Figure 2:**
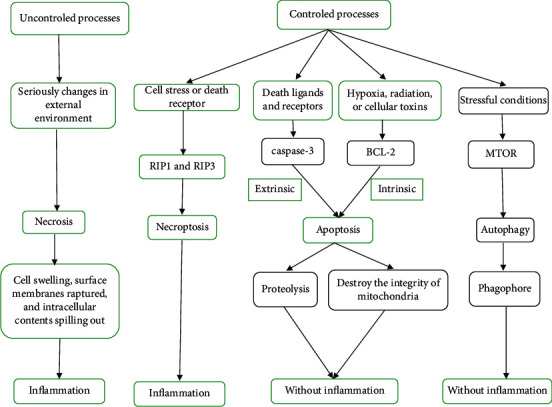
Cell death modalities in I/R injury. Different mechanisms of cell death: necrosis is characterized as cell and organelle swelling; subsequently, the surface membranes ruptured, and intracellular contents spilled out. Necrotic cells induce intensive immune stimulation, which lead to cell infiltration and cytokine release, infiltrate inflammatory cells, and generate cytokines. Necroptosis is defined as programmed necrosis, which shares similar features with necrosis. Apoptosis is programmed cell death, and it is less immunostimulatory than necrosis. The mechanisms of apoptosis include two major pathways: intrinsic and extrinsic pathways. Autophagy is the main way of cells to disposal of protein aggregates and damaged organelles.

I/R injury-induced tissue injury includes two portions: ischemia injury and reperfusion injury. When ischemia progresses, metabolites accumulated, and metabolic acidosis occurred. If the blood supply is restored, the increased inflammation and ROS production aggregated injury. If the damage is slight, the function of cells may activate the recovery system to maintain their function and survival. However, if the injury is severe, cells will die through the apoptotic or necrotic pathway [68]. Different ways of cell death through various pathogenesis (Figure 2).

I/R induces cell death via various mechanisms, including necrosis, necroptosis, apoptosis, and autophagy [65]. Necrosis is characterized as cell and organelle swelling [69]; subsequently, the surface membranes ruptured, and intracellular contents spilled out [65]. Necrotic cells induce intensive immune stimulation, which lead to inflammatory cell infiltration and cytokine release. If the cells encounter excessive stress, necrosis occurred [70]. The progression of necrosis induces serious changes in the external environment, which are induced by chemical, biological, or physical injury. Necrosis is usually considered to be random and uncontrolled processes, in which the cell responses to overwhelming stress. Necroptosis is termed to be programmed necrosis [71]. It occurs in pathologic states, especially I/R injury. Necroptosis shares similar features with necrosis. Necroptosis is activated by cell stress or death receptors, such as TNF receptor-1 and Fas receptor. The combination of death receptors and ligands leads to mobilization and activation of a group of receptor-interacting protein kinases (RIKs). RIP1 and RIP3 are members of the receptor-interacting protein kinase (RIPK) family. The formation of the necrotic complex between RIP1 and RIP3 can mediate caspase-independent cell necrosis [72, 73]. Overexpressed RIP3 may induce upexpression of both ROS and Ca^2+^ and enhance NF-*κ*B protein regulation [74]. Low-expressed RIP3 may suppress apoptosis [69, 75]. The activation of RIP3 occurs in TNF-induced necroptosis. There is an association between necroptosis and inflammation in the pathogenesis of I/R injury. So, the research on the association may be useful to understand the mechanism and provide guidance for treatment.

Apoptosis is programmed cell death, characterized as shrinkage of cells and nuclei, with plasma membrane integrity persisted. It is less immunostimulatory than necrosis. The mechanisms of apoptosis include two major pathways: intrinsic and extrinsic pathways. Extrinsic pathway is the death receptor pathway, activated by death ligands and receptors such as TNF-*α*, tumor necrosis factor-related weak inducer of apoptosis (TWeAK), Fas ligand, tumor necrosis factor (TNF) related apoptosis-inducing ligand (TRAIL), and TL1A [76–79]. These complexes may induce to cleave caspase-3 and subsequently kill cells through proteolysis in injured cells [80]. Intrinsic pathway is a mitochondrial pathway, activated by hypoxia, cellular toxins, and radiation. This progress involves B-cell lymphoma-2 (Bcl-2) protein family members, including Bax and BaK [81, 82]. These prodeath proteins transport proapoptotic proteins from the intermembrane to the outer membrane by activating the permeability of the membrane [83]. Subsequently, prodeath proteins bind to the apoptotic protease-activating factor-1 (APAF1) and assemble the apoptosome; then, the complex activates caspase-3 and -9, inducing cellular protein cleavage [69]. Bcl-2 proteins are activated and unregulated and accumulated on mitochondrial membranes of ischemic cells [84–87]. Ischemia needs oxidative stress, evoked by reperfusion, to activate Bcl-2 proteins. Numerous apoptogenic factors are released including cytochrome c, caspase activator Omi, high-temperature-required protein A2 (HtrA2), second mitochondria-derived activator of caspases (Smac), and direct inhibitor of apoptosis protein (IAP) binding protein with low pI (DIABLO), but their roles and whether their inhibitors could be used for I/R injury are unclear.

Autophagy is the main mechanism of cells to disposal of damaged protein aggregates and cellular toxins [69, 88]. It may provide survival mechanism of cells to resistance of stressful conditions, such as infection, hypoxia, and mitochondrial dysfunction. However, if autophagy is out of control, it will lead to death of cells. In the process of autophagy, biological macromolecules and damaged organelles in the cytoplasm will be degraded in membrane vesicles. Autophagy involves cytoplasmic components and ruptured organelles. This process can be activated by I/R injury [89]. The main regulator of autophagy is the mammalian target of rapamycin (mTOR). mTOR will be inactivated in stress or nutrition deficiency. Inactivated mTOR will inhibit the formation of phagophores. The extension of the autophagic vesicle requires the participation of the autophagy-related protein 8/light chain 3 (Atg8/LC3) complex and Atg12-Atg5-Atg16 complex. Autophagy might upregulate the survival rate of cells. The inhibition of autophagy may amplify I/R injury [90–92]. However, if the injury is severe, cellwould be deregulated by autophagy. [93–95]. Autophagy begins with assembly of phagophore. [96, 97]. Vesicular autophagosome is formed by phagophore expansion to fully encase the cell constituents. Autophagy is regulated by mTOR. However, other regulatory mechanisms of autophagy need to be further investigated.

By interrupting the cell death process, cell survival rate can be increased, and the recovery time for lower limb function can be reduced, which may be effective ways to reduce I/R injury.

## 5. Treatment of Traditional Chinese Medicine

TCM has been applied in China and some other Asian nations for more than 2000 years. Several studies have shown that TCM can be used in the treatment of I/R injury. TCM may reduce I/R injury through angiogenesis, antioxidant effect, reducing oxidative stress, inhibiting inflammatory cytokines' release, and so on.

In the mouse hindlimb ischemia model, hydroxysafflor yellow A enhances blood flow recovery and increases capillary and arteriole densities. This indicated that hydroxysafflor yellow A could promote angiogenesis [98]. Another research study found that tubeimoside-I (TBM) promoted endothelial cell viability, migration, and tube formation in human umbilical vein endothelial cells. In the hindlimb ischemia model, TBM may promote angiogenesis [99].

**Figure 3:**
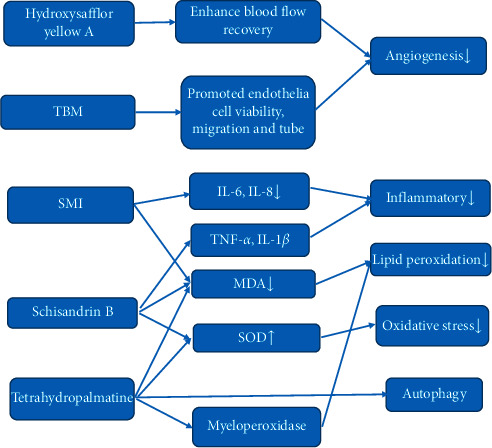
Hydroxysafflor yellow A enhances blood flow recovery and increases capillary and arteriole densities. This indicated that hydroxysafflor yellow A could promote angiogenesis. Tubeimoside-I (TBM) may promote angiogenesis by using the hindlimb ischemia model. If the patient pretreated in Shengmai injection (SMI) before applying automatic gas-filled tourniquet, their IL-6, IL-8, and MDA levels would be significantly decreased. This indicated that SMI may attenuate lipid peroxidation and systemic inflammatory response. Schisandrin B reduces MDA, increases SOD activity, and attenuates plasma inflammatory cytokines. These suggested that schisandrin B reduced I/R injury of the skeletal muscle by attenuating oxidative stress and inflammation. Study on tetrahydropalmatine showed that it may reduce myeloperoxidase and MDA, increase SOD, and inhibit autophagy.

IL-6, IL-8, and plasma malondialdehyde (MDA) were used to indicate lipid peroxidation and systemic inflammatory response. If the patient pretreated with Shengmai injection (SMI) before applying automatic gas-filled tourniquet, their IL-6, IL-8, and MDA levels would be significantly decreased. This indicated that SMI may attenuate lipid peroxidation and systemic inflammatory response [100]. Schisandrin B may ameliorate ischemia histological changes of the skeletal muscle. In addition, schisandrin B reduces MDA, increases SOD activity, and attenuates plasma inflammatory cytokines. These suggested that schisandrin B reduced I/R injury of the skeletal muscle by attenuation oxidative stress and inflammation [101]. Study on tetrahydropalmatine showed that it may reduce myeloperoxidase and MDA, increase SOD, and inhibit autophagy (Figure 3).

I/R injury results from the complex pathophysiology process, which links to multiple mechanisms; any treatment targeting single link is insufficient to resolve this disease. Current studies provide abundant evidence on the mechanisms of TCM in I/R injury. However, most studies focus on single compound, extracted from Chinese herbs. In fact, most TCMs are used together to form a formula. TCM formula has advantages that may affect multiple targets, which may enhance efficacy and attenuate toxicity. The interactions between different components need further research, which may effectively explore the network of TCM formula. This may be another important research direction. In addition, TCM will be pretreated by decocting or other methods before use, which is an important part of TCM treatment. Nevertheless, its effect on TCM is reported scarcely.

## 6. Conclusion

I/R injury is an important clinical problem in PAD; it is still a critical challenge for doctors. Mitochondria play a central role in I/R injury on account of cell signaling, oxidative stress, energy production, and cell damage. The cell death pathways rely on the degree of injury and the microenvironment. However, the mechanisms of I/R injury are complex and include various aspects. An enhanced understanding of the pathophysiology and cell death pathways is critical for new therapies. In addition, TCM has been used to treat diseases for a long time. Recent research has verified the potential utility of TCM for the treatment of I/R injury. However, the mechanisms and combination of TCMs need further research.

## References

[1] Crawford F., Welch K., Andras A., Chappell F. M. (2016). Ankle brachial index for the diagnosis of lower limb peripheral arterial disease. *The Cochrane Database of Systematic Reviews*.

[2] Aronow W. S., Ahn C. (1994). Prevalence of coexistence of coronary artery disease, peripheral arterial disease, and atherothrombotic brain infarction in men and women ≥62 years of age. *The American Journal of Cardiology*.

[3] Hiatt W. R., Hoag S., Hamman R. F. (1995). Effect of diagnostic criteria on the prevalence of peripheral arterial disease. *Circulation*.

[4] Criqui M. H., Ninomiya J. K., Wingard D. L., Ji M., Fronek A. (2008). Progression of peripheral arterial disease predicts cardiovascular disease morbidity and mortality. *Journal of the American College of Cardiology*.

[5] Kurian G. A., Rajagopal R., Vedantham S., Rajesh M. (2016). The role of oxidative stress in myocardial ischemia and reperfusion injury and remodeling: revisited. *Oxidative Medicine and Cellular Longevity*.

[6] Eliason J. L., Wakefield T. W. (2009). Metabolic consequences of acute limb ischemia and their clinical implications. *Seminars in Vascular Surgery*.

[7] Yassin M. M. I., Harkin D. W., Barros D’Sa A. A. B., Halliday M. I., Rowlands B. J. (2002). Lower limb ischemia-reperfusion injury triggers a systemic inflammatory response and multiple organ dysfunction. *World Journal of Surgery*.

[8] Galluzzi L., Kepp O., Trojel-Hansen C., Kroemer G. (2012). Mitochondrial control of cellular life, stress, and death. *Circulation Research*.

[9] Tao M., You C.-P., Zhao R.-R. (2014). Animal mitochondria: evolution, function, and disease. *Current Molecular Medicine*.

[10] Wang J., Toan S., Zhou H. (2020). New insights into the role of mitochondria in cardiac microvascular ischemia/reperfusion injury. *Angiogenesis*.

[11] Ferguson R. A., Ball D., Krustrup P. (2001). Muscle oxygen uptake and energy turnover during dynamic exercise at different contraction frequencies in humans. *The Journal of Physiology*.

[12] Charles A.-L., Lejay A., Zoll J., Chakfe N., Geny B. (2015). Re: “protective effect of focal adhesion kinase against skeletal muscle reperfusion injury after acute limb ischemia”. *European Journal of Vascular and Endovascular Surgery*.

[13] Flück M., von Allmen R. S., Ferrié C., Tevaearai H., Dick F. (2015). Protective effect of focal adhesion kinase against skeletal muscle reperfusion injury after acute limb ischemia. *European Journal of Vascular and Endovascular Surgery*.

[14] Pipinos I. I., Judge A. R., Selsby J. T. (2007). The myopathy of peripheral arterial occlusive disease: part 1. Functional and histomorphological changes and evidence for mitochondrial dysfunction. *Vascular and Endovascular Surgery*.

[15] Pipinos I. I., Sharov V. G., Shepard A. D. (2003). Abnormal mitochondrial respiration in skeletal muscle in patients with peripheral arterial disease. *Journal of Vascular Surgery*.

[16] Pipinos I. I., Judge A. R., Zhu Z. (2006). Mitochondrial defects and oxidative damage in patients with peripheral arterial disease. *Free Radical Biology and Medicine*.

[17] Yang L., Guo Y., Fan X. (2020). Amelioration of coagulation disorders and inflammation by hydrogen-rich solution reduces intestinal ischemia/reperfusion injury in rats through NF-*κ*B/NLRP3 pathway. *Mediators of Inflammation*.

[18] Barbieri E., Sestili P. (2012). Reactive oxygen species in skeletal muscle signaling. *Journal of Signal Transduction*.

[19] Lu B., Kwan K., Levine Y. A. (2014). *α*7 nicotinic acetylcholine receptor signaling inhibits inflammasome activation by preventing mitochondrial DNA release. *Molecular Medicine*.

[20] Thaveau F., Zoll J., Rouyer O. (2007). Ischemic preconditioning specifically restores complexes I and II activities of the mitochondrial respiratory chain in ischemic skeletal muscle. *Journal of Vascular Surgery*.

[21] Thaveau F., Zoll J., Bouitbir J. (2009). Contralateral leg as a control during skeletal muscle ischemia-reperfusion. *Journal of Surgical Research*.

[22] Pang C. Y., Yang R. Z., Zhong A., Xu N., Boyd B., Forrest C. R. (1995). Acute ischaemic preconditioning protects against skeletal muscle infarction in the pig. *Cardiovascular Research*.

[23] Walker P. M. (1991). Ischemia/reperfusion injury in skeletal muscle. *Annals of Vascular Surgery*.

[24] Bushell A. J., Klenerman L., Davies H., Grierson I., McArdle A., Jackson M. J. (2002). Ischaemic preconditioning of skeletal muscle 2. Investigation of the potential mechanisms involved. *The Journal of Bone and Joint Surgery. British Volume*.

[25] Kuzon W. M., Walker P. M., Mickle D. A. G., Harris K. A., Pynn B. R., McKee N. H. (1986). An isolated skeletal muscle model suitable for acute ischemia studies. *Journal of Surgical Research*.

[26] Moses M. A., Addison P. D., Neligan P. C. (2005). Inducing late phase of infarct protection in skeletal muscle by remote preconditioning: efficacy and mechanism. *American Journal of Physiology-Regulatory, Integrative and Comparative Physiology*.

[27] Troitzsch D., Moosdorf R., Hasenkam J. M., Nygaard H., Vogt S. (2013). Effects of cyclosporine pretreatment on tissue oxygen levels and cytochrome oxidase in skeletal muscle ischemia and reperfusion. *Shock*.

[28] Tupling R., Green H., Senisterra G., Lepock J., McKee N. (2001). Effects of ischemia on sarcoplasmic reticulum Ca2+ uptake and Ca2+ release in rat skeletal muscle. *American Journal of Physiology-Endocrinology and Metabolism*.

[29] Hagberg H. (1985). Intracellular pH during ischemia in skeletal muscle: relationship to membrane potential, extracellular pH, tissue lactic acid and ATP. *Pflügers Archiv: European Journal of Physiology*.

[30] Martou G., O’Blenes C. A., Huang N. (2006). Development of an in vitro model for study of the efficacy of ischemic preconditioning in human skeletal muscle against ischemia-reperfusion injury. *Journal of Applied Physiology (1985)*.

[31] Welsh D. G., Lindinger M. I. (1997). Metabolite accumulation increases adenine nucleotide degradation and decreases glycogenolysis in ischaemic rat skeletal muscle. *Acta Physiologica Scandinavica*.

[32] Powers S. K., Murlasits Z., Wu M., Kavazis A. N. (2007). Ischemia-reperfusion-induced cardiac injury: a brief review. *Medicine & Science in Sports & Exercise*.

[33] Ivanics T., Miklós Z., Ruttner Z. (2000). Ischemia/reperfusion-induced changes in intracellular free Ca2+ levels in rat skeletal muscle fibers—an in vivo study. *Pflügers Archiv—European Journal of Physiology*.

[34] Di Lisa F., Canton M., Carpi A. (2011). Mitochondrial injury and protection in ischemic pre- and postconditioning. *Antioxidants & Redox Signaling*.

[35] Tran T. P., Tu H., Liu J., Muelleman R. L., Li Y.-L. (2012). Mitochondria-derived superoxide links to tourniquet-induced apoptosis in mouse skeletal muscle. *PLoS One*.

[36] Baudry N., Laemmel E., Vicaut E. (2008). In vivo reactive oxygen species production induced by ischemia in muscle arterioles of mice: involvement of xanthine oxidase and mitochondria. *American Journal of Physiology-Heart and Circulatory Physiology*.

[37] Tupling R., Green H., Senisterra G., Lepock J., McKee N. (2001). Effects of 4-h ischemia and 1-h reperfusion on rat muscle sarcoplasmic reticulum function. *American Journal of Physiology-Endocrinology and Metabolism*.

[38] Korthuis R. J., Granger D. N. (1993). Reactive oxygen metabolites, neutrophils, and the pathogenesis of ischemic-tissue/reperfusion. *Clinical Cardiology*.

[39] Gissel H. (2005). The role of Ca2+ in muscle cell damage. *Annals of the New York Academy of Sciences*.

[40] Wang W. Z., Fang X.-H., Stephenson L. L., Khiabani K. T., Zamboni W. A. (2008). Ischemia/reperfusion-induced necrosis and apoptosis in the cells isolated from rat skeletal muscle. *Journal of Orthopaedic Research*.

[41] Ueta C. B., Gomes K. S., Ribeiro M. A., Mochly-Rosen D., Ferreira J. C. B. (2017). Disruption of mitochondrial quality control in peripheral artery disease: new therapeutic opportunities. *Pharmacological Research*.

[42] Youle R. J., van der Bliek A. M. (2012). Mitochondrial fission, fusion, and stress. *Science*.

[43] Chen G. Y., Nuñez G. (2010). Sterile inflammation: sensing and reacting to damage. *Nature Reviews Immunology*.

[44] Iyer S. S., Pulskens W. P., Sadler J. J. (2009). Necrotic cells trigger a sterile inflammatory response through the Nlrp3 inflammasome. *Proceedings of the National Academy of Sciences*.

[45] McDonald B., Pittman K., Menezes G. B. (2010). Intravascular danger signals guide neutrophils to sites of sterile inflammation. *Science*.

[46] Grenz A., Homann D., Eltzschig H. K. (2011). Extracellular adenosine: a safety signal that dampens hypoxia-induced inflammation during ischemia. *Antioxidants & Redox Signaling*.

[47] Petzelbauer P., Zacharowski P. A., Miyazaki Y. (2005). The fibrin-derived peptide B*β*15-42 protects the myocardium against ischemia-reperfusion injury. *Nature Medicine*.

[48] Powers K. A., Szászi K., Khadaroo R. G. (2006). Oxidative stress generated by hemorrhagic shock recruits toll-like receptor 4 to the plasma membrane in macrophages. *Journal of Experimental Medicine*.

[49] Arbour N. C., Lorenz E., Schutte B. C. (2000). TLR4 mutations are associated with endotoxin hyporesponsiveness in humans. *Nature Genetics*.

[50] Dai H., Thomson A. W., Rogers N. M. (2019). Dendritic cells as sensors, mediators, and regulators of ischemic injury. *Frontiers in Immunology*.

[51] Frye C. C., Bery A. I., Kreisel D., Kulkarni H. S. (2021). Sterile inflammation in thoracic transplantation. *Cellular and Molecular Life Sciences*.

[52] Oliveira T. H. C. d., Marques P. E., Proost P., Teixeira M. M. M. (2018). Neutrophils: a cornerstone of liver ischemia and reperfusion injury. *Laboratory Investigation*.

[53] Fan Q., Tao R., Zhang H. (2019). Dectin-1 contributes to myocardial ischemia/reperfusion injury by regulating macrophage polarization and neutrophil infiltration. *Circulation*.

[54] Xu L., Sharkey D., Cantley L. G. (2019). Tubular GM-CSF promotes late MCP-1/CCR2-mediated fibrosis and inflammation after ischemia/reperfusion injury. *Journal of the American Society of Nephrology*.

[55] Sakai K., Nozaki Y., Murao Y. (2019). Protective effect and mechanism of IL-10 on renal ischemia-reperfusion injury. *Laboratory Investigation*.

[56] Bagchi A. K., Surendran A., Malik A., Jassal D. S., Ravandi A., Singal P. K. (2020). IL-10 attenuates OxPCs-mediated lipid metabolic responses in ischemia reperfusion injury. *Scientific Reports*.

[57] Kreisel D., Sugimoto S., Tietjens J. (2011). Bcl3 prevents acute inflammatory lung injury in mice by restraining emergency granulopoiesis. *Journal of Clinical Investigation*.

[58] Ricklin D., Hajishengallis G., Yang K., Lambris J. D. (2010). Complement: a key system for immune surveillance and homeostasis. *Nature Immunology*.

[59] Kulik L., Fleming S. D., Moratz C. (2009). Pathogenic natural antibodies recognizing annexin IV are required to develop intestinal ischemia-reperfusion injury. *The Journal of Immunology*.

[60] Kanzler H., Barrat F. J., Hessel E. M., Coffman R. L. (2007). Therapeutic targeting of innate immunity with toll-like receptor agonists and antagonists. *Nature Medicine*.

[61] Fenhammar J., Rundgren M., Forestier J., Kalman S., Eriksson S., Frithiof R. (2011). Toll-like receptor 4 inhibitor TAK-242 attenuates acute kidney injury in endotoxemic sheep. *Anesthesiology*.

[62] Diepenhorst G. M. P., van Gulik T. M., Hack C. E. (2009). Complement-mediated ischemia-reperfusion injury: lessons learned from animal and clinical studies. *Annals of Surgery*.

[63] Investigators A. A., Armstrong P. W., Granger C. B. (2007). Pexelizumab for acute ST-elevation myocardial infarction in patients undergoing primary percutaneous coronary intervention: a randomized controlled trial. *JAMA*.

[64] Shernan S. K., Fitch J. C. K., Nussmeier N. A. (2004). Impact of pexelizumab, an anti-C5 complement antibody, on total mortality and adverse cardiovascular outcomes in cardiac surgical patients undergoing cardiopulmonary bypass. *The Annals of Thoracic Surgery*.

[65] Moser M., Nieswandt B., Ussar S., Pozgajova M., Fässler R. (2008). Kindlin-3 is essential for integrin activation and platelet aggregation. *Nature Medicine*.

[66] Müller F., Mutch N. J., Schenk W. A. (2009). Platelet polyphosphates are proinflammatory and procoagulant mediators in vivo. *Cell*.

[67] Levi M., van der Poll T. (2010). Inflammation and coagulation. *Critical Care Medicine*.

[68] Weissmüller T., Campbell E. L., Rosenberger P. (2008). PMNs facilitate translocation of platelets across human and mouse epithelium and together alter fluid homeostasis via epithelial cell-expressed ecto-NTPDases. *Journal of Clinical Investigation*.

[69] Wang J., Toan S., Zhou H. (2020). Mitochondrial quality control in cardiac microvascular ischemia-reperfusion injury: new insights into the mechanisms and therapeutic potentials. *Pharmacological Research*.

[70] Cai Z., Manalo D. J., Wei G. (2003). Hearts from rodents exposed to intermittent hypoxia or erythropoietin are protected against ischemia-reperfusion injury. *Circulation*.

[71] Fantacci M., Bianciardi P., Caretti A. (2006). Carbamylated erythropoietin ameliorates the metabolic stress induced in vivo by severe chronic hypoxia. *Proceedings of the National Academy of Sciences*.

[72] Geng L., Zhang G., Yao M., Fang Y. (2020). Rip 1-dependent endothelial necroptosis participates in ischemia-reperfusion injury of mouse flap. *Journal of Dermatological Science*.

[73] Yang R., Hu K., Chen J. (2017). Necrostatin-1 protects hippocampal neurons against ischemia/reperfusion injury via the RIP3/DAXX signaling pathway in rats. *Neuroscience Letters*.

[74] Zhu P., Hu S., Jin Q. (2018). Ripk3 promotes ER stress-induced necroptosis in cardiac IR injury: a mechanism involving calcium overload/XO/ROS/mPTP pathway. *Redox Biology*.

[75] Zhou H., Zhu P., Guo J. (2017). Ripk3 induces mitochondrial apoptosis via inhibition of FUNDC1 mitophagy in cardiac IR injury. *Redox Biology*.

[76] Xiao G., Lyu M., Wang Y. (2019). Ginkgo flavonol glycosides or ginkgolides tend to differentially protect myocardial or cerebral ischemia-reperfusion injury via regulation of TWEAK-fn14 signaling in heart and brain. *Frontiers in Pharmacology*.

[77] Sanz A. B., Ruiz-Andres O., Sanchez-Niño M. D., Ruiz-Ortega M., Ramos A. M., Ortiz A. (2016). Out of the TWEAKlight: elucidating the role of Fn14 and TWEAK in acute kidney injury. *Seminars in Nephrology*.

[78] Lu Y., Xi J., Zhang Y. (2020). MicroRNA-214-5p protects against myocardial ischemia reperfusion injury through targeting the FAS ligand. *Archives of Medical Science*.

[79] Jiang Y., Chen X., Fan M. (2017). TRAIL facilitates cytokine expression and macrophage migration during hypoxia/reoxygenation via ER stress-dependent NF-*κ*B pathway. *Molecular Immunology*.

[80] Wu M.-Y., Yiang G.-T., Liao W.-T. (2018). Current mechanistic concepts in ischemia and reperfusion injury. *Cellular Physiology and Biochemistry*.

[81] Birdsall H. H., Green D. M., Trial J. (1997). Complement C5a, TGF-*β*1, and MCP-1, in sequence, induce migration of monocytes into ischemic canine myocardium within the first one to five hours after reperfusion. *Circulation*.

[82] Whelan R. S., Kaplinskiy V., Kitsis R. N. (2010). Cell death in the pathogenesis of heart disease: mechanisms and significance. *Annual Review of Physiology*.

[83] Zhou H., Toan S., Zhu P., Wang J., Ren J., Zhang Y. (2020). DNA-PKcs promotes cardiac ischemia reperfusion injury through mitigating BI-1-governed mitochondrial homeostasis. *Basic Research in Cardiology*.

[84] Diwan A., Krenz M., Syed F. M. (2007). Inhibition of ischemic cardiomyocyte apoptosis through targeted ablation of Bnip3 restrains postinfarction remodeling in mice. *Journal of Clinical Investigation*.

[85] Ji X., Luo Y., Ling F. (2007). Mild hypothermia diminishes oxidative DNA damage and pro-death signaling events after cerebral ischemia: a mechanism for neuroprotection. *Frontiers in Bioscience*.

[86] Weinbroum A. A., Hochhauser E., Rudick V. (1997). Direct induction of acute lung and myocardial dysfunction by liver ischemia and reperfusion. *The Journal of Trauma: Injury, Infection, and Critical Care*.

[87] Wu B., Qiu W., Wang P. (2007). p53 independent induction of PUMA mediates intestinal apoptosis in response to ischaemia-reperfusion. *Gut*.

[88] Wang J., Zhou H. (2020). Mitochondrial quality control mechanisms as molecular targets in cardiac ischemia-reperfusion injury. *Acta Pharmaceutica Sinica B*.

[89] Ma S., Wang Y., Chen Y., Cao F. (2015). The role of the autophagy in myocardial ischemia/reperfusion injury. *Biochimica et Biophysica Acta (BBA)—Molecular Basis of Disease*.

[90] Cardinal J., Pan P., Tsung A. (2009). Protective role of cisplatin in ischemic liver injury through induction of autophagy. *Autophagy*.

[91] Carloni S., Girelli S., Scopa C., Buonocore G., Longini M., Balduini W. (2010). Activation of autophagy and Akt/CREB signaling play an equivalent role in the neuroprotective effect of rapamycin in neonatal hypoxia-ischemia. *Autophagy*.

[92] Huang C., Liu W., Perry C. N. (2010). Autophagy and protein kinase C are required for cardioprotection by sulfaphenazole. *American Journal of Physiology-Heart and Circulatory Physiology*.

[93] Hamacher-Brady A., Brady N. R., Logue S. E. (2007). Response to myocardial ischemia/reperfusion injury involves Bnip3 and autophagy. *Cell Death & Differentiation*.

[94] Hariharan N., Zhai P., Sadoshima J. (2011). Oxidative stress stimulates autophagic flux during ischemia/reperfusion. *Antioxidants & Redox Signaling*.

[95] Matsui Y., Kyoi S., Takagi H. (2008). Molecular mechanisms and physiological significance of autophagy during myocardial ischemia and reperfusion. *Autophagy*.

[96] Gottlieb R. A., Gustafsson Å. B. (2011). Mitochondrial turnover in the heart. *Biochimica et Biophysica Acta (BBA)—Molecular Cell Research*.

[97] Lejay A., Fang F., John R. (2016). Ischemia reperfusion injury, ischemic conditioning and diabetes mellitus. *Journal of Molecular and Cellular Cardiology*.

[98] Chen T., Chen N., Pang N. (2016). Hydroxysafflor yellow A promotes angiogenesis via the angiopoietin 1/tie-2 signaling pathway. *Journal of Vascular Research*.

[99] Yang X., Li X., Luo M. (2021). Tubeimoside I promotes angiogenesis via activation of eNOS-VEGF signaling pathway. *Journal of Ethnopharmacology*.

[100] Wang L., Wang W., Zhao X. (2011). Effect of Shenmai injection, a traditional Chinese medicine, on pulmonary dysfunction after tourniquet-induced limb ischemia-reperfusion. *Journal of Trauma: Injury, Infection & Critical Care*.

[101] Zhu N., Cai C., Zhou A., Zhao X., Xiang Y., Zeng C. (2017). Schisandrin B prevents hind limb from ischemia-reperfusion-induced oxidative stress and inflammation via MAPK/NF-*κ*B pathways in rats. *BioMed Research International*.

